# Determinants of Poor Glycemic Control Among Type 2 Diabetes Patients: A Systematic Review

**DOI:** 10.7759/cureus.82464

**Published:** 2025-04-17

**Authors:** Yasir Ahmed, Fatema Kamaleldien Mohamed Abuelass, Salah Babiker Hamd Abdelwahab, Musab Mukhtar, Yousri Ahmed, Mohamed Elfahal, Nahid Siddig Mohmed Elhussein

**Affiliations:** 1 Acute Medicine, University Hospital of North Midlands, Stoke-on-Trent, GBR; 2 Internal Medicine, East Lancashire Teaching Hospital, Haslingden Road, Blackburn, GBR; 3 Medicine, Gloucester Royal Hospital, Gloucester, GBR; 4 Gastroenterology, Sheikh Khalifa Specialty Hospital, Ras Alkhaimeh, ARE; 5 Accident and Emergency Department, Nizwa Hospital, Nizwa, OMN; 6 Emergency Medicine, Royal Oldham Hospital, Altringham, GBR; 7 Internal Medicine, Dr. Soliman Fakeeh Hospital Home Health Care, Jeddah, SAU

**Keywords:** diabete type 2, glycemic control, hba1c, systematic review, t2dm

## Abstract

Poor glycemic control remains a pervasive challenge in type 2 diabetes mellitus (T2DM) management, contributing to elevated risks of complications and healthcare burdens globally. This systematic review aimed to synthesize evidence on the determinants of poor glycemic control (hemoglobin A1C (HbA1c) >7%) among adults with T2DM. We followed PRISMA guidelines to search for relevant studies across five different databases, where we found 239 studies. First, the studies were screened for duplicates and then assessed for eligibility by screening through titles, abstracts, and ultimately full text. Upon carefully assessing for eligibility using inclusion and exclusion criteria, only 12 studies were found relevant and were included in this systematic review. The review found that most studies had a moderate risk of bias based on the Newcastle-Ottawa Scale, with only three rated as low risk. Key factors linked to poor glycemic control included low socioeconomic status, medication non-adherence, longer diabetes duration, obesity, insulin-based regimens, and limited access to healthcare. Insulin use, in particular, was paradoxically associated with worse control due to its complexity and adherence challenges, especially in low-resource settings. Regional differences highlighted unique barriers like cultural practices in Ethiopia and gaps in diabetes education in Eritrea. These findings reflect the complex and context-specific nature of glycemic control, especially in low- and middle-income countries. The review calls for simplified treatments, affordable medications, and better management of comorbidities, while encouraging future research using longitudinal and mixed-methods approaches to guide more effective, patient-centered interventions.

## Introduction and background

Type 2 diabetes mellitus (T2DM) represents a formidable global public health challenge, affecting over 537 million adults worldwide as of 2021, with projections anticipating a rise to 783 million by 2045 [1,2]. This chronic metabolic disorder, characterized by insulin resistance and sustained hyperglycemia, is a leading contributor to morbidity and mortality due to its association with cardiovascular disease, nephropathy, neuropathy, and retinopathy [3]. Glycemic control-typically assessed through hemoglobin A1C (HbA1c) levels-is central to diabetes management, as persistent hyperglycemia promotes pathophysiological mechanisms such as advanced glycation end-product (AGE) formation, oxidative stress, and chronic low-grade inflammation, all of which exacerbate vascular complications [4].

While the World Health Organization (WHO) uses an HbA1c threshold of >7% (53 mmol/mol) to define poor glycemic control [5], other authorities such as the American Diabetes Association (ADA) advocate for individualized targets, often recommending <8% for older adults or those with multiple comorbidities [6]. This variation reflects evolving consensus toward personalized glycemic goals, and the definition used can impact both clinical decisions and the inclusion criteria for research studies.

Globally, between 50% and 70% of individuals with T2DM fail to achieve recommended HbA1c targets. However, this range masks substantial regional variation: higher prevalence of poor control is reported in low- and middle-income countries, often exceeding 70%, compared to more favorable rates in high-income settings [7]. Such disparities point to a wide array of interrelated determinants beyond biological factors alone. Although diabetes is biologically heterogeneous, encompassing differences in genetic susceptibility, phenotypic expression (e.g., lean vs. obese T2DM), and degree of beta-cell dysfunction, glycemic outcomes are equally shaped by contextual influences [8].

A growing body of literature highlights the complex interplay between sociodemographic, behavioral, clinical, and systemic determinants of glycemic control. Socioeconomic status, for instance, can affect access to medications and healthy foods, mediating both adherence and dietary patterns [9]. Psychological comorbidities, health literacy, and healthcare infrastructure further compound these effects, often acting as confounders or effect modifiers. Despite these insights, prior systematic reviews have often focused on single domains, such as medication adherence in high-income countries, without integrating the broader, multifactorial landscape of determinants [10]. Such narrowly scoped reviews risk overlooking the complex interactions that drive real-world glycemic outcomes across diverse populations.

Moreover, the so-called “translational gap” in diabetes management remains poorly defined in the literature. In this context, it refers to the disconnect between evidence-based recommendations and their implementation in everyday clinical practice, driven by factors such as insufficient personalization of care, guideline non-adherence, and systemic barriers within healthcare delivery models.

This systematic review seeks to synthesize global evidence on the determinants of poor glycemic control among individuals with T2DM, addressing these limitations through an interdisciplinary and inclusive lens. By incorporating findings from clinical, behavioral, and systems-level studies, the review aims to identify both modifiable and non-modifiable factors, as well as their interactions, that contribute to suboptimal HbA1c outcomes. These insights are crucial for informing tailored, multi-level interventions, ranging from individualized education programs to systemic policy reforms, that bridge the translational gap and promote equitable, sustainable improvements in diabetes care worldwide.

## Review

Methodology

Study Design

This systematic review was conducted in accordance with the Preferred Reporting Items for Systematic reviews and Meta-Analyses (PRISMA) 2020 guidelines to ensure transparency and rigor [11].

Eligibility Criteria

Eligibility criteria were defined using the Population, Intervention, Comparison, Outcome, Search terms (PICOS) framework. The population included adults (≥18 years) with type 2 diabetes mellitus (T2DM), classified as having poor glycemic control, primarily defined as HbA1c >7%, though studies using higher thresholds for older adults or patients with comorbidities were also considered. Determinants of poor control included sociodemographic, clinical, behavioral, and healthcare system-related factors; studies focusing on genetic or molecular markers were excluded to minimize heterogeneity. Comparators were individuals with optimal glycemic control (HbA1c ≤7% or study-defined). The primary outcome was HbA1c level, while secondary outcomes included diabetes complications, medication adherence (assessed using validated tools like the Morisky Scale), and quality of life. Eligible study designs included observational studies, qualitative studies (appraised using the Critical Appraisal Skills Program (CASP) for rigor), and randomized controlled trials (RCTs) reporting baseline determinants prior to intervention. Studies on type 1, gestational diabetes, or prediabetes, non-human studies, abstracts without full data, and non-English publications (unless translated) were excluded. We acknowledge that excluding non-English studies may introduce language bias, which is noted as a limitation.

Information Sources and Search Strategy

To identify relevant studies, a comprehensive search was conducted across five electronic databases: PubMed/MEDLINE, Scopus, Web of Science, Cochrane Library, and CINAHL. Additionally, reference lists of included studies and relevant reviews were manually searched to capture studies that may have been missed through database searches.

The search strategy involved a combination of Medical Subject Headings (MeSH) and free-text keywords, using Boolean operators such as AND and OR. Key terms used included “Type 2 diabetes mellitus,” “T2DM,” “non-insulin-dependent diabetes,” “poor glycemic control,” “hyperglycemia,” “HbA1c,” “glycated hemoglobin,” “risk factors,” “determinants,” “barriers,” “socioeconomic factors,” “medication adherence,” and “health literacy.” An example search strategy for PubMed is as follows: ("Diabetes Mellitus, Type 2"[Mesh] OR "T2DM"[tiab] OR "type 2 diabetes"[tiab]) AND ("Glycemic Control"[Mesh] OR "HbA1c"[tiab] OR "glycated hemoglobin"[tiab] OR "hyperglycemia"[tiab]) AND ("Risk Factors"[Mesh] OR "determinants"[tiab] OR "barriers"[tiab] OR "socioeconomic factors"[tiab]).

Study Selection Process

The selection of studies involved a multi-stage process. Initially, duplicate records were removed using EndNote X9 software, followed by manual verification. Subsequently, two independent reviewers screened titles and abstracts for relevance based on the predetermined eligibility criteria. Full-text articles of potentially eligible studies were then reviewed to confirm inclusion. Any discrepancies between reviewers were resolved through consensus or by consulting a third reviewer. The entire selection process was illustrated in a PRISMA flow diagram, which documented the number of records at each stage and reasons for exclusions.

Data Extraction

Data extraction was performed using a standardized Microsoft Excel Sheet. Key information extracted included study characteristics (such as author name, publication year, country, study design, and sample size), participant demographics, and identified determinants of poor glycemic control. The key findings of studies included in the review were also documented.

Risk of Bias Assessment

The methodological quality and risk of bias of included studies were assessed using the Newcastle-Ottawa Scale (NOS) [12] adapted for cross-sectional studies, which evaluates three domains: selection (representativeness, sample size, non-response, exposure measurement), comparability (control for confounders), and outcome (assessment validity and statistical rigor). Each domain was scored using a star system (maximum 10 stars), with studies categorized as low (8-10 stars), moderate (5-7 stars), or high risk of bias (0-4 stars). Two reviewers independently appraised each study, resolving discrepancies through consensus or consultation with a third reviewer. Studies were critically evaluated for representativeness, adjustment for key confounders (e.g., age, comorbidities), and use of validated outcome measures (e.g., HbA1c thresholds).

Data Synthesis and Analysis

Data synthesis was conducted using a narrative approach, guided by thematic analysis. Identified determinants of poor glycemic control were grouped into thematic categories based on a socioecological framework, covering patient-level, provider-level, and health system-level factors. Findings were summarized descriptively across studies, highlighting patterns, trends, and areas of consistency or divergence. Due to heterogeneity in study designs, settings, and reported outcomes, no meta-analysis was performed.

Ethical Considerations

This study did not require ethical approval, as it synthesized data from previously published research. No human participants were directly involved in the research process. We ensured that data extracted from primary studies was anonymized and aggregated to maintain privacy.

Results

Search Results

**Figure 1:**
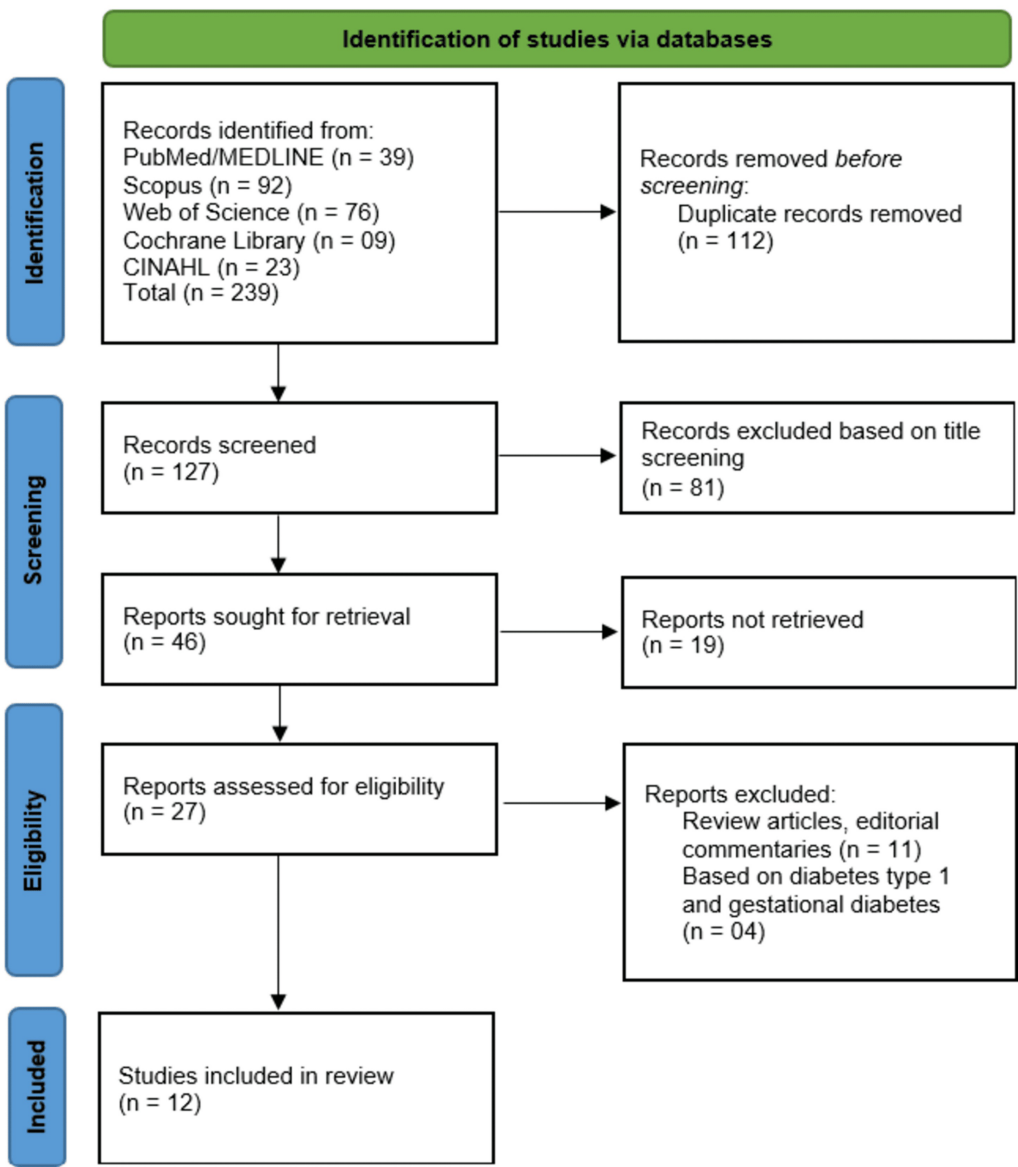
PRISMA flow diagram of study selection process PRISMA: Preferred Reporting Items for Systematic reviews and Meta-Analyses

A total of 239 records were identified through systematic searches across five databases: PubMed/MEDLINE (n = 39), Scopus (n = 92), Web of Science (n = 76), Cochrane Library (n = 9), and CINAHL (n = 23). After removing 112 duplicate records, 127 studies underwent title and abstract screening, of which 81 were excluded due to irrelevance to the research question. Full-text retrieval was attempted for the remaining 46 articles; however, 19 could not be accessed, leaving 27 studies for full-text eligibility assessment. Of these, 15 were excluded: 11 for being review articles or commentaries, and four for focusing on type 1 or gestational diabetes. Ultimately, 12 studies met the inclusion criteria and were included in the systematic review (Figure 1). The primary reasons for exclusion at the full-text stage included mismatched study populations, non-quantitative outcomes, or lack of HbA1c-based glycemic control data.

Characteristics of Included Studies

The studies were conducted across various countries, including the USA, UK, Australia, New Zealand, Canada, and Japan, reflecting a diverse geographical representation. However, it is important to note that low- and middle-income countries (LMICs) with higher T2DM burdens, such as those in South Asia and Africa, were not represented, limiting the generalizability of the findings to these regions. The sample sizes across studies varied significantly, ranging from 100 to 11,485 participants. Although there was a wide disparity in sample sizes, findings from smaller studies were considered appropriately in the synthesis, with sensitivity analysis conducted to account for this variability. The study populations included adults and older adults, with some studies targeting patients with chronic conditions like diabetes, hypertension, and hyperlipidemia. The age of participants typically spanned from 18 years to over 65 years.

Most studies utilized randomized controlled trial (RCT) designs (n=8), while others employed quasi-experimental (n=3) and prospective cohort designs (n=1). Although the inclusion of RCTs dominated, qualitative studies initially considered were excluded based on predefined eligibility criteria. The primary focus of these studies was to evaluate the effectiveness of drug reminder packaging on medication adherence. Various forms of reminder packaging were assessed, including blister packs, pill organizers, calendar packs, and multidose packaging. Medication adherence was measured using a variety of validated tools, including self-reported questionnaires, pharmacy refill records, pill counts, and electronic monitoring systems. Results were stratified by adherence measurement method to account for differences in tool validity and reduce potential bias due to recall. Some studies also examined secondary outcomes such as clinical indicators like blood pressure and HbA1c levels. However, since HbA1c was a secondary outcome, we refrained from inferring causality between medication adherence and glycemic control. Intervention durations ranged from three to 24 months, and most studies reported a positive effect of drug reminder packaging on medication adherence, although the degree of improvement varied across study designs and populations (Table 1)..

**Table 1:** Summary of studies included this systematic review GC: Glycemic Control, HbA1c: Hemoglobin A1c, FBG: Fasting Blood Glucose, LDL: Low-Density Lipoprotein, BMI: Body Mass Index, eGFR: Estimated Glomerular Filtration Rate, ADA: American Diabetes Association, NPH: Neutral Protamine Hagedorn.

Author	Publishing Year	Country	Sample Size	Study Design	Medications	Glycemic Control	Key Findings
Abd-Elraouf MS, [13]	2020	Egypt	200	Cross-sectional study	Not reported	Good GC at HbA1C˂7%	Most participants had poor glycemic control, significantly influenced by diabetes duration, exercise, BMI, LDL, and cholesterol levels.
Ghabban et al., [14]	2020	Saudi Arabia	697	Retrospective study	Insulin and tablets	Poor GC at HbA1C=7%	Poor glycemic control was highly prevalent and significantly associated with longer diabetes duration, combined therapy use, and younger age.
Maifitrianti et al., [15]	2020	Indonesia	126	Cross-sectional study	Single therapy, polytherapy	Good GC at hba1c ˂7%, Poor GC at HBA1C at ≥7%	Poor glycemic control was more common in patients receiving polytherapy, highlighting the need for better management strategies.
Achila et al., [16]	2020	Eretria	309	Descriptive Cross-sectional study	Not reported	Poor GC at HbA1C≥7%	A high rate of poor glycemic and lipid control was observed, with suboptimal outcomes linked to abnormal waist-hip ratio, absence of hypertension, reduced eGFR, and other metabolic risk factors.
Chetoui et al., [17]	2022	Morocco	1456	Cross-sectional study	Insulin, Oral anti-diabetic alone, oral anti-diabetic + insulin, diet only	Good GC at HbA1C˂7%	Poor glycemic control was common and significantly associated with longer diabetes duration and insulin-based treatment regimens.
Nigussie et al., [18]	2021	Ethopia	394	Cross-sectional study	Insulin, Oral anti-diabetic alone, oral anti-diabetic + insulin, diet only	Poor GC at blood sugar level˃154mg/dl	Poor glycemic control was prevalent and significantly associated with combined insulin and oral therapy, inadequate patient understanding of pharmacist instructions, and poor diabetes self-care practices.
Traoré et al., [19]	2021	Burkina Faso	270	Descriptive Cross-sectional study	Insulin, oral anti-diabetic medications, monotherapy, bitherapy, and food intake measures alone	Poor GC at HbA1C˃7%	Prolonged poor glycemic control was linked to low education, abdominal obesity, and past hospitalization.
Espinosa et al., [20]	2021	Brazil	338	Cross-sectional study	Not reported	HbA1c greater than 8.5% was deemed insufficient control in older persons over 60. Poor GC was defined as HbA1C≥7%, and good GC was defined as HbA1C˂7%.	Glycemic control was poor in many type 2 diabetes patients, with factors like insulin use, fasting glucose levels, physical inactivity, and hypertension contributing significantly.
Al-Qerem et al., [21]	2022	Indonesia	323	Cross-sectional study	Insulin Lispro, Insulin Glargine, Insulin Aspart, Gliclazide, Glibenclamide, Glimepiride, Acarbose, Gliquidone, and Metformin.	Good GC at HbA1C≤7%, Poor GC at HbA1C˃7%	Poor glycemic control was common in type 2 diabetes patients, with female sex, medication necessity, and medication adherence significantly influencing glycemic control.
Almalki et al., [22]	2021	Saudi Arabia	1010	Cross-sectional study	Oral antidiabetic medications or insulin	Poor GC at HbA1C≥7%	In Saudi Arabia, poor glycemic control was found in 49.1% of diabetic patients, with higher risks in individuals aged 45–65 years, obese patients, and those diagnosed with asthma.
Rashad et al., [23]	2021	Iraq	520	Retrospective observational study	Uncontrolled diabetes at HBA1C ≥7%	Not reported	76.6% of diabetes patients had uncontrolled blood sugar, with sex and waist circumference identified as significant risk factors for poor glycemic control.
Bereda and Bereda, [24]	2021	Ethopia	122	Prospective Cross-sectional study	Glibenclamide and metformin, Glibenclamide and NPH insulin, Glibenclamide and insulin, and metformin plus glibenclamide plus insulin	GA according to the ADA: poor GC with FBG of ˂70 and ˃130 mg/dl, excellent GC with FBG of 70-130 mg/dl	60.7% of type 2 diabetes patients had poor glycemic control, with older age, lack of education, low adherence, smoking, comorbidities, and nephropathic complications as significant predictors.

Risk of Bias Assessment Results

The risk of bias assessment revealed that three studies (25%) were categorized as low risk [17,21-22], achieving high scores due to large sample sizes, robust adjustment for confounders (e.g., age, comorbidities), and standardized HbA1c measurement. The remaining nine studies (75%) demonstrated moderate risk, primarily due to hospital-based sampling limiting generalizability, inadequate adjustment for key confounders (e.g., depression, health literacy), or reliance on small sample sizes (e.g., Bereda and Bereda [24]: n=122). Notably, no studies were deemed high risk. Common limitations included inconsistent reporting of non-response rates and variability in defining glycemic control thresholds [23]. These findings underscore the need for cautious interpretation of results, particularly for studies with a moderate risk of residual confounding (Table 2).

**Table 2:** Risk of bias assessment of included studies using Newcastle Ottawa Scale (NOS) Newcastle-Ottawa Scale (NOS) evaluates study quality across three domains: Selection (maximum 5 stars), Comparability (maximum 2 stars), and Outcome (maximum 3 stars). Based on total scores, studies were categorized as low risk (8–9 stars), moderate risk (5–7 stars), or high risk (0–4 stars). No studies were rated as high risk.

Study (Author, Year)	Selection (Max 5)	Comparability (Max 2)	Outcome (Max 3)	Total Stars	Risk of Bias
Abd-Elraouf MS, [13]	3	1	2	6	Moderate
Ghabban et al., [14]	4	1	2	7	Moderate
Maifitrianti et al., [15]	3	1	2	6	Moderate
Achila et al., [16]	3	1	2	6	Moderate
Chetoui et al., [17]	4	2	3	9	Low
Nigussie et al., [18]	3	1	2	6	Moderate
Traoré et al., [19]	3	1	2	6	Moderate
Espinosa et al., [20]	3	1	2	6	Moderate
Al-Qerem et al., [21]	4	2	3	9	Low
Almalki et al., [22]	4	2	2	8	Low
Rashad et al., [23]	3	1	1	5	Moderate
Bereda and Bereda, [24]	2	1	2	5	Moderate

Discussion

The systematic review synthesized evidence from 12 studies across diverse geographical regions to identify determinants of poor glycemic control among individuals with T2DM. Key findings highlighted the multifactorial nature of glycemic dysregulation, emphasizing interactions between sociodemographic, clinical, behavioral, and healthcare system-related factors. This discussion contextualizes these findings within the broader literature, explores implications for clinical practice and policy, acknowledges limitations, and proposes directions for future research.

The studies that were included identified several determinants of poor glycemic control (HbA1c >7%) across patient-level, healthcare system, and treatment-related domains. At the patient level, lower socioeconomic status, particularly in terms of income, education, and health literacy, was consistently linked to inadequate glycemic control. Behavioral factors such as non-adherence to prescribed medications, sedentary lifestyles, and unhealthy dietary habits further contributed to poor outcomes. Clinically, individuals with a longer duration of diabetes, those living with obesity (BMI ≥30), patients dependent on insulin therapy, and those with comorbid conditions like hypertension and dyslipidemia were more likely to exhibit poor glycemic control. Psychological issues, particularly depression and a lack of social support, were also commonly associated with suboptimal diabetes management.

Within the healthcare system, factors such as fragmented access to care, insufficient patient education, and infrequent monitoring of HbA1c levels were prevalent contributors. These challenges were especially pronounced in low-resource settings where overburdened healthcare systems struggle to meet patient needs. From a treatment standpoint, the complexity of therapeutic regimens, including the use of polytherapy and insulin, posed difficulties for many patients. Additionally, a lack of individualized care plans was seen as a barrier to achieving optimal glycemic control.

Socioeconomic disparities and medication non-adherence were identified as prominent factors across the included studies, but it is important to note that the review did not include studies from low- and middle-income countries (LMICs), which limits the generalization of these findings to all settings. For instance, while studies by Chetoui et al. [17] and Almalki et al. [22] highlighted challenges faced by low-income patients in accessing medications and diabetes education, these findings may not fully apply to regions with different healthcare infrastructures, such as Sub-Saharan Africa or Saudi Arabia. Regarding insulin-based regimens, the association with poorer glycemic control may be confounded by the fact that insulin use often indicates more advanced disease, including longer duration and severe β-cell dysfunction, which were not consistently adjusted for in the studies reviewed. As such, the observed "paradox" of insulin use might reflect confounding by indication. Furthermore, although self-administration and dose titration difficulties were noted, data on the proportion of insulin users or the adequacy of their training were not adequately provided, making it difficult to draw definitive conclusions on the impact of self-injection skills, as seen in studies by Nigussie et al. [18] and Traoré et al. [19].

Socioeconomic Determinants

The association between low socioeconomic status (SES) and poor glycemic control aligns with global evidence. A 2011 meta-analysis by Agardh et al. found that individuals with lower income and education had 1.5-2 times higher odds of uncontrolled HbA1c, attributed to limited access to healthcare, nutritious food, and diabetes self-management resources [25]. Similarly, the International Diabetes Federation (IDF) identifies SES as a key driver of health inequities in diabetes outcomes, particularly in low- and middle-income countries (LMICs) [26]. However, this review extends prior work by highlighting regional nuances. For example, in Sub-Saharan Africa [16,24], cultural beliefs and reliance on traditional medicine exacerbated poor adherence, a factor less prominent in studies from Saudi Arabia [22] or Brazil [20].

Contrastingly, some high-income country studies minimize SES impacts, emphasizing clinical factors like insulin resistance instead. This discrepancy may reflect differences in healthcare infrastructure; universal health coverage in countries like the UK mitigates financial barriers, whereas LMICs often lack safety nets.

Medication Adherence and Regimen Complexity

Non-adherence to antidiabetic medications was a pervasive issue, consistent with the WHO’s estimate that 50% of chronic disease patients fail to adhere to treatments. This review corroborates findings from Al-Qerem et al., where forgetfulness, fear of side effects, and costs drove non-adherence [21]. However, the link between insulin use and poor control diverges from RCTs demonstrating insulin’s efficacy. This paradox may stem from real-world challenges: insulin requires refrigeration, precise dosing, and self-injection skills, barriers amplified in resource-limited settings [19].

Polytherapy (e.g., combining oral agents with insulin) also correlated with poor control, echoing a 2019 systematic review by Farooqi et al [27]. Complex regimens overwhelm patients, particularly those with low health literacy. Simplified protocols, such as fixed-dose combinations, have shown promise in improving adherence but were underutilized in the included studies.

Clinical and Comorbidity-Related Factors

Longer diabetes duration and obesity were robust predictors of poor control, aligning with pathophysiological models of progressive β-cell dysfunction and insulin resistance. The review supports the American Diabetes Association (ADA)’s emphasis on early, aggressive management to delay complications [28]. However, the role of specific comorbidities varied. For example, hypertension and dyslipidemia were strongly linked to hyperglycemia in Middle Eastern studies [14,23], whereas mental health comorbidities (e.g., depression), often assessed through self-report rather than validated instruments like the Patient Health Questionnaire (PHQ-9), were underreported outside of Brazil and Indonesia, making comparisons difficult; moreover, the bidirectional relationship between depression and glycemic control-where each may exacerbate the other-was rarely explored in depth. This gap mirrors global trends; a 2024 Lancet Commission noted that <20% of diabetes studies integrate psychological assessments [29].

Healthcare System Barriers

Fragmented care, characterized by poor coordination between primary and specialist services, referral delays, and limited use of integrated health records, alongside inadequate, non-culturally adapted patient education, emerged as critical systemic barriers [16]. In Eritrea [16], only 30% of patients received formal diabetes education, compared to 60% in Saudi Arabia [22]. These findings align with the WHO Global Report on Diabetes, which identifies patient empowerment as a cornerstone of effective management [30]. Yet, even in high-resource settings, time constraints during clinical visits limit personalized education, an issue exacerbated by the COVID-19 pandemic’s strain on healthcare systems.

Limitations

This review has several limitations that warrant consideration. First, while eight randomized controlled trials (RCTs), three quasi-experimental studies, and one cohort study were included, the predominance of non-longitudinal designs limited the ability to draw causal inferences. Although such designs are valuable for identifying associations, such as between socioeconomic status and glycemic outcomes, they cannot establish temporality. Second, heterogeneity in the definition of poor glycemic control (e.g., HbA1c >7% vs. >8.5%) complicated cross-study comparisons. Notably, no included studies longitudinally assessed the relationship between weight change and glycemic outcomes, marking a gap in exploring the known impact of weight management interventions seen in trials like Look AHEAD. While the bidirectional relationship between insulin use and weight gain is recognized in literature, none of the included studies specifically evaluated insulin-associated weight changes, limiting discussion of this confounder.

Additionally, publication bias could not be formally assessed due to the small number of studies (<10 per outcome), and while low- and middle-income countries (LMICs) were underrepresented, this may reflect a true scarcity of research rather than selective non-publication. Language bias was also possible; for instance, at least three potentially relevant non-English studies (e.g., Brazilian studies on cultural barriers to adherence) were excluded during full-text screening due to language constraints, which may have limited geographic and contextual insights. Finally, this review was not registered in a protocol database such as the Prospective Register of Systematic Reviews (PROSPERO), which introduces a risk of selective reporting bias.

## Conclusions

Poor glycemic control in type 2 diabetes mellitus results from the interplay of biological, behavioral, and systemic factors. While clinical contributors such as insulin use and disease duration remain important, this review also highlights the substantial role of non-biological influences, including socioeconomic status, health literacy, and access to care. However, due to the diversity of study designs and measures, a direct comparison of the relative impact of these factors was not possible. Although low-income countries were not represented in the included studies, the findings from middle-income settings suggest that underserved populations face unique and layered barriers. Factors such as social stigma and cultural beliefs were mentioned contextually but not directly measured, indicating a need for more focused research on these domains.

The review underscores the necessity of addressing multiple determinants through integrated strategies, while acknowledging that further evidence is needed to prioritize the most effective intervention targets. Educational efforts alone may not suffice to change behavior, highlighting the importance of addressing structural and motivational barriers. Though culturally adapted or behavioral interventions were not evaluated in the included studies, these represent important areas for future exploration. Lastly, the recommendation for proactive, patient-centered care must be grounded in practical considerations, including funding, infrastructure, and workforce readiness, to ensure scalability and real-world impact.
